# Validation of Bedaquiline Phenotypic Drug Susceptibility Testing Methods and Breakpoints: a Multilaboratory, Multicountry Study

**DOI:** 10.1128/JCM.01677-19

**Published:** 2020-03-25

**Authors:** Koné Kaniga, Akio Aono, Emanuele Borroni, Daniela Maria Cirillo, Christel Desmaretz, Rumina Hasan, Lavania Joseph, Satoshi Mitarai, Sadia Shakoor, Gabriela Torrea, Nazir Ahmed Ismail, Shaheed V. Omar

**Affiliations:** a Johnson & Johnson Global Public Health, Titusville, New Jersey, USA; b Department of Mycobacterium Reference and Research, The Research Institute of Tuberculosis, Japan Anti-tuberculosis Association, Kiyose, Japan; c Emerging Bacterial Pathogens Unit, IRCCS San Raffaele Scientific Institute, Milan, Italy; d Department of Biomedical Sciences, Mycobacteriology Unit, Institute of Tropical Medicine, Antwerp, Belgium; e Department of Pathology and Laboratory Medicine, The Aga Khan University, Karachi, Pakistan; f Faculty of Infectious and Tropical Disease, London School of Hygiene and Tropical Medicine, London, United Kingdom; g Center for Tuberculosis, National and WHO Supranational TB Reference Laboratory, National Institute for Communicable Diseases, National Health Laboratory Services, Johannesburg, South Africa; h Department of Medical Microbiology, University of Pretoria, Pretoria, South Africa; i Department of Internal Medicine, University of Witwatersrand, Johannesburg, South Africa; University of North Carolina School of Medicine

**Keywords:** bedaquiline, drug resistance, drug susceptibility testing, *Mycobacterium tuberculosis*, tuberculosis, variants

## Abstract

Drug-resistant tuberculosis persists as a major public health concern. Alongside efficacious treatments, validated and standardized drug susceptibility testing (DST) is required to improve patient care. This multicountry, multilaboratory external quality assessment (EQA) study aimed to validate the sensitivity, specificity, and reproducibility of provisional bedaquiline MIC breakpoints and World Health Organization interim critical concentrations (CCs) for categorizing clinical Mycobacterium tuberculosis isolates as susceptible/resistant to the drug. Three methods were used: Middlebrook 7H11 agar proportion (AP) assay, broth microdilution (BMD) assay, and mycobacterial growth indicator tube (MGIT) assay. Each of the five laboratories tested the 40-isolate (20 unique isolates, duplicated) EQA panel at three time points. The study validated the sensitivity and specificity of a bedaquiline MIC susceptibility breakpoint of 0.12 μg/ml for the BMD method and WHO interim CCs of 1 μg/ml for MGIT and 0.25 μg/ml for the 7H11 AP methods. Categorical agreements between observed and expected results and sensitivities/specificities for correctly identifying an isolate as susceptible/resistant were highest at the 0.25, 0.12, and 1 μg/ml bedaquiline concentrations for the AP method, BMD (frozen or dry plates), and MGIT960, respectively. At these concentrations, the very major error rates for erroneously categorizing an isolate as susceptible when it was resistant were the lowest and within CLSI guidelines. The most highly reproducible bedaquiline DST methods were MGIT960 and BMD using dry plates. These findings validate the use of standardized DST methodologies and interpretative criteria to facilitate routine phenotypic bedaquiline DST and to monitor the emergence of bedaquiline resistance.

## Abstract (precis)

Drug-resistant tuberculosis persists as a major public health concern. Alongside efficacious treatments, validated and standardized drug susceptibility testing (DST) is required to improve patient care. This multicountry, multilaboratory external quality assessment (EQA) study aimed to validate the sensitivity, specificity, and reproducibility of provisional bedaquiline MIC breakpoints and World Health Organization interim critical concentrations (CCs) for categorizing clinical Mycobacterium tuberculosis isolates as susceptible/resistant to the drug.

## INTRODUCTION

Drug-resistant tuberculosis (TB) is a major public health concern. In 2017, 3.5% of new Mycobacterium tuberculosis cases and 18% of previously treated cases were cases of rifampin-resistant TB (RR-TB) and/or multidrug-resistant TB (MDR-TB) (1). Additionally, 8.5% of MDR-TB cases were estimated to be extensively drug resistant (XDR) (1). It is thus important to have a range of effective anti-TB drugs against both drug-resistant strains and wild-type strains (1). In addition to treatment, widespread uptake and usage of validated and standardized drug susceptibility testing (DST) and rapid molecular diagnostic tests are required to optimize care in patients with MDR-TB, especially in low-income countries, where the burden of TB and drug-resistant disease is high (2).

Bedaquiline (BDQ) is a diarylquinoline antimycobacterial agent that acts differently from other anti-TB agents through inhibiting ATP synthase, leading to ATP depletion and decreased mycobacterial survival; it also has bactericidal and sterilizing properties (3). In the treatment of MDR-TB, outcomes have considerably improved with the use of BDQ-based regimens (4–7). A number of resistance-associated variants (RAVs) that may decrease susceptibility to BDQ have been reported. Characterized RAVs include mutations in BDQ target gene *atpE* (3), efflux pump regulator gene *Rv0678* (8–10), gene *pepQ* (11, 12), and gene *Rv1979c* (12). *atpE* RAVs that reduce BDQ activity have been observed previously *in vitro* (13) but rarely in patient clinical isolates (14, 15). In contrast, mutations in *Rv0678* have led to low-level resistance in isolates obtained both *in vitro* and in the clinic (15). Currently, the clinical relevance of the *pepQ* and *Rv1979c* RAVs is unclear (15).

Susceptible isolates may be considered resistant to BDQ based purely on the presence of an *Rv0678* RAV, despite the variety of RAVs in *Rv0678* and their differing effects on BDQ MICs (10). Moreover, an association between specific RAVs and either BDQ MICs or clinical outcome has not been established to date; also, sufficient knowledge to correctly interpret whole-genome sequencing data is not available.

In the absence of reliable, rapid, and robust molecular or genotypic BDQ DST methods, phenotypic DST of BDQ should be used to guide treatment of patients with MDR-TB requiring BDQ as part of their treatment regimen and/or to monitor the development of resistance to BDQ during therapy. A combination of this test with whole-genome sequencing data would be valuable for drug resistance surveillance purposes (16). The two currently established approaches for TB DST are the proportion method using critical concentration (CC) data and the MIC-based method. The CC (or antimicrobial susceptibility testing breakpoint) is the lowest concentration of the drug that inhibits 99% (90% for pyrazinamide) of wild-type M. tuberculosis strains, not including clinical strains classified as resistant (17, 18). As this automatically classifies 5% of wild-type strains as drug resistant, CC results in misclassification of resistant and susceptible strains (18). Additionally, as combination treatment is mandatory for TB, the use of clinical outcome data for single drugs is impractical (18). The MIC is the lowest concentration that completely inhibits M. tuberculosis growth *in vitro* (19). For BDQ phenotypic DST, Middlebrook 7H10 (7H10) or Middlebrook 7H11 (7H11) agar dilution and Middlebrook 7H9 (7H9) broth microdilution (BMD) MIC determination methods have been developed and validated in a multicountry, multilaboratory study (20). The BMD method provided DST results from pure cultures after an incubation period of 14 days or less, whereas agar media required 21 days or more. In a clinical setting, however, an additional period of 2 to 6 weeks is needed to obtain pure colonies for preparing the inoculum used in either method (20).

A liquid-based phenotypic DST with a faster turnaround, such as the mycobacterial growth indicator tube (MGIT) assay (Becton, Dickinson, NJ, USA), would be more efficient to guide therapy, as previously reported for BDQ (21, 22). The World Health Organization (WHO) lists MGIT as the preferred reference DST method for BDQ using an interim CC of 1 μg/ml. The agar proportion (AP) method using an interim CC of 0.25 μg/ml is also recommended (17, 23). Additionally, provisional BDQ MIC breakpoints of 1 μg/ml and 0.12 μg/ml for the MGIT and BMD methods, respectively, have been reported (15). Rancoita et al. have previously shown the reliability of bedaquiline testing using microdilution with microtiter plates (24).

The objective of this external quality assessment (EQA) study was to validate the sensitivity, specificity, and reproducibility of the WHO interim CCs and of the provisional BDQ MIC breakpoints (15) for identifying clinical M. tuberculosis isolates as susceptible or resistant to BDQ using three methods: MGIT, 7H11 AP, and BMD.

## MATERIALS AND METHODS

### Participating laboratories.

Ten WHO TB Supranational Reference Laboratory Network (SRLN) members were invited to participate in the study. Five laboratories (Lab-1, Japan; Lab-2, Pakistan; Lab-3, South Africa; Lab-4, Italy; Lab-5, Belgium) were selected on the basis of their willingness to participate, availability of resources, and adequate profiles of isolates required for further analysis.

In this study (TMC207-TBCECOFF), investigators were blind to each other. The sponsor and principal investigator laboratory were not blind to the other laboratories participating in the study due to contractual and logistic considerations. Using a specific data collection form, all laboratories sent their raw data set directly to the principal investigator, who performed the final analyses; data were shared with the sponsor after the data had been analyzed.

### EQA panel.

Three populations of isolates were used in the EQA panel. The first population included wild-type (for BDQ) M. tuberculosis clinical isolates from the South African National Institute for Communicable Diseases (NICD). The second population consisted of genotypically characterized *Rv0678*, *atpE* RAVs (laboratory engineered at the Institute of Tropical Medicine [Antwerp, Belgium]) (Table 1), and the third population consisted of genotypically characterized *Rv0678*, *atpE* and dual-*Rv0678*/*atpE* RAVs (laboratory engineered at NICD [South Africa]) (Table 1). The second and third populations were expected to be BDQ resistant (confirmed at the MIC and MGIT960 breakpoints [15]) and to be resistant at the WHO interim CCs and were used as reference strains for the study.

**TABLE 1:** Genotypic characterization of BDQ mutant isolates used in the EQA panel

Site	Isolate ID^*a*^	Gene target(s)	Nucleotide mutation	Amino acid mutation
Institute of Tropical Medicine (Antwerp, Belgium)	1	*atpE*	G187C	Ala63Pro
	2	*Rv0678*	T276A	Tyr92STOP
	3	*Rv0678*	C403T	Ala131Trp

South African National Institute for Communicable Diseases	1	*Rv0678*/*atpE*	201_206delCAGCAC/A83C	Ser68_Thr69del/Asp28Ala
	2	*Rv0678*	T131C	Leu44Pro

a ID, identifier.

In total, the EQA panel comprised 40 M. tuberculosis isolates (20 unique strains in duplicate) that were uniquely barcoded and labeled in a blind manner with respect to sites (with the exclusion of the South Africa researchers who prepared the panel; however, the operators remained blind at this site). Among the 20 unique strains, 14 were BDQ-susceptible clinical isolates (for which whole-genome sequencing and DST data were available), 4 (BDQEQA2017006, BDQEQA2017010, BDQEQA2017026, and BDQEQA2017018) were well-characterized *in vitro*-derived *atpE* or *Rv0678* mutants, 1 (BDQEQA2017040) was a dual *Rv0678* and *atpE* mutant (although the *atpE* RAV in this strain showed no effect on BDQ susceptibility), and 1 (BDQEQA2017039) was the quality control (QC) strain M. tuberculosis H37Rv. Each laboratory tested the 40-isolate EQA panel at three time points, using three independently prepared inocula, on separate dates by three phenotypic DST methods in parallel. For initial propagation, each laboratory used its own H37Rv reference strain as a control for each test method.

### 7H11 agar proportion method.

For the 7H11 AP method, laboratories were provided with BDQ active pharmaceutical ingredient (lot number A17HB1824; Beerse, Belgium). BDQ-containing agar medium was prepared at three BDQ concentrations (0.25, 0.5, and 1 μg/ml) using a stock solution of 1 mg/ml made in dimethyl sulfoxide, with adjustments made according to the conversion factor of 1.2 for the fumarate salt. Standard Middlebrook 7H11 base and oleic acid albumin dextrose enrichment were used to prepare all drug-containing media, and polystyrene petri dishes and/or tubes were used to prepare 7H11 media. Inocula of culture media were standardized in all experiments. The undiluted (10°) M. tuberculosis suspension, measured to a McFarland standard of 1, was subjected to mixing, and 0.1 ml was transferred to 0.9 ml in the ﬁrst dilution tube (10^−1^ dilution) (∼5 × 10^6^ CFU/ml). Working suspensions were made using a 10-fold dilution of this M. tuberculosis suspension with sterile deionized water or saline solution, achieving a 10^−2^ dilution which was then used for inoculation. Inoculated plates were incubated at 37°C and checked for contamination after 1 week followed by DST reading performed between week 4 and week 8 (25). The percentage of resistant bacteria was calculated as the number of colonies on the drug divided by the number of colonies on the control × 100; if this proportion was ≥1%, the strain was considered resistant to the tested drug. Susceptibility/resistance was determined either by calculation or by visual comparison of the growth observed on the drug-containing plate with that observed on the control plates without an optical aid. An isolate was considered susceptible to BDQ if no growth was observed on the drug-containing plates or if the growth observed on the drug-containing plate was less than the growth observed on the most highly diluted control tube (10^−3^, representing 1% of the level of possible growth). If the growth level observed on the drug-containing tube was equal to or greater than the growth level observed on the most highly diluted control plate, the isolate was considered resistant to BDQ.

### MGIT960 DST method.

In the current study, the Bactec MGIT 960 DST methodology was followed as previously detailed (22, 26), with minor modifications for BDQ. Laboratories were provided with lyophilized BDQ vials containing 170 μg/vial potency-adjusted dimethyl sulfoxide (Becton, Dickinson and Company).

BDQ-containing media were prepared at two BDQ concentrations (1 μg/ml and 2 μg/ml). In brief, a MGIT960 growth supplement for DST was used in the MGIT960 system (Becton, Dickinson). The procedure was the standard protocol recommended for DST of first-line drugs by the use of built-in software. Lyophilized BDQ was reconstituted with 2.0 ml filter-sterilized dimethyl sulfoxide per vial, and appropriate drug stock (0.1 or 0.2 ml) was added per drug tube. If cultures were 3 to 5 days old, bacterial suspensions were prepared from MGIT subcultures as recommended by the manufacturer (MGIT manual) (27). Inoculated drug-containing MGIT960 tubes were placed in a DST three-tube-set carrier or captured using BD EpiCentre TBExist software, placed in the MGIT960 instrument, and incubated at 37°C (±1°C) for a maximum of 28 days. As the MGIT system is automated, the instrument continuously reads all tubes, using fluorescent sensors to measure growth unit (GU) levels at 60-min intervals for a maximum of 28 days. When the control reached a growth unit value of 400 between day 4 and day 28, the instrument flagged the DST set as “complete.” Bacteria were defined as resistant if the growth unit value of the drug-containing tube was >100 and the growth unit value of the growth control tube was ≥400 (28).

### MIC determination by the 7H9 broth microdilution method.

The 7H9 BMD MIC was determined in accordance with Clinical and Laboratory Standards Institute (CLSI) reference method M7-A10 with M. tuberculosis focus (20, 27) by the use of frozen and dry microtiter plates (Thermo Fisher Scientific Inc., Waltham, MA, USA) containing BDQ and 11 other drugs used to treat TB. Concentrations and QC ranges of the drugs on the plates were detailed previously by Kaniga et al. (27).

Frozen microtiter plates were prepared with BDQ serial dilutions in 2× oleic acid-albumin-dextrose-catalase (OADC)-supplemented 7H9 medium (7H9) at 2× final drug concentrations. Isolates were grown on 7H11 agar medium or Löwenstein-Jensen medium, and the colonies were resuspended in saline solution-Tween with glass beads (TF, USA) to prepare a concentration of a McFarland standard of 1 corresponding theoretically to ∼5 × 10^7^ CFU/ml for M. tuberculosis. If required, additional colonies were added or sterile deionized water was used to adjust the M. tuberculosis suspension to a level equivalent to a McFarland standard of 1. A 2× inoculum of M. tuberculosis isolates was prepared by adding 255 μl of the suspension (McFarland standard of 1) to 12.5 ml sterile deionized water (50-fold dilution from the McFarland standard of 1) to give 1 × 10^6^ CFU/ml. The 2× inoculum was transferred into a disposable inoculum reservoir for manual pipetting or used directly on an autoinoculator (Thermo Fisher, USA), and then 100 μl was transferred to the microtiter plate wells. The final inoculum size targeted in the plates was 5 × 10^5^ CFU/ml, and the final BDQ concentrations were 4, 2, 1, 0.5, 0.25, 0.12, 0.06, 0.03, 0.015, and 0.008 μg/ml and the control.

Dry microtiter plates were prepared with BDQ serial dilutions containing 1× drugs. Approximately 1 to 3 loopfuls (10-μl loops) of mycobacterial colonies, cultured on solid media no older than 28 days, were transferred into saline solution-Tween with glass beads (TF, USA) to prepare a suspension representing a McFarland standard of 1. A 100-fold dilution of the McFarland standard 1 was made by transferring 100 μl of the suspension into the tube containing 10-ml Middlebrook 7H9 with OADC (TF, USA), and the diluted inoculum was subjected to vortex mixing for ∼30 s. A 100-μl volume of the resultant suspension was then inoculated into each well as described for the frozen plate. The target inoculum size was 5 × 10^5^ CFU/ml.

For both formats of the plates, once inoculated, the isolates were incubated at a temperature of 36°C (±1°C) for the 14-day duration. The inoculum was used as the positive-growth control well for the entire plate. Microtiter plates were read according to laboratory procedures at day 14 postinoculation.

### Quality control.

For all test methods performed, all laboratories were required to test a susceptible laboratory control strain with each batch of isolates tested. In addition, the laboratory QC strain was included in the EQA panel and processed in a blind manner. Results for the batches tested were considered valid if the laboratory results passed QC testing.

### Statistical methods.

Analyses included use of the Kappa statistic for agreement and further determination of the sensitivity, specificity, and categorical agreement data for each test method (7H11 AP, MGIT960, and BMD MIC) at each critical concentration tested. The sensitivity value represented the percentage classified as resistant by the test method against the total number of true resistant isolates, and the specificity value represented the percentage classified as susceptible against the total number of true susceptible isolates. The levels of intra- and interlaboratory reproducibility were assessed for all isolates using the provisional CC/BP data and by resistance subtype. Intralaboratory reproducibility was measured, and if all three EQA panel isolate replicates tested in agreement, the assay was classified as reproducible. Isolates with a missing replicate (no result) were excluded from the analysis. Interlaboratory reproducibility was measured by calculating the percentage of agreement for each EQA strain for all testing laboratories, and the average agreement was calculated to determine the reproducibility as a percentage (all isolates were included irrespective of whether replicate values were missing). Errors representing results showing resistance by the evaluated method and susceptibility by the reference standard were defined as major errors, and errors representing results showing susceptibility by the evaluated method and resistance by the reference standard were defined as very major errors (29). The final validated critical concentration for BDQ DST was chosen based on the overall aspects, which included the highest sensitivity/specificity/categorical agreement and the lowest error rates, ensuring that these fell within the boundaries of the CLSI guidelines.

### Data availability.

The data sharing policy of Janssen Pharmaceutical Companies of Johnson & Johnson is available at https://www.janssen.com/clinical-trials/transparency. As noted on that site, requests for access to the study data can be submitted through the Yale Open Data Access (YODA) Project site at http://yoda.yale.edu.

## RESULTS

### Categorical agreement, sensitivity and specificity, and error rates.

Analyses of the overall sensitivity, specificity, categorical agreement, and error rates for all isolates are presented irrespective of laboratory or replicate. For the AP, BMD (frozen plates), BMD (dry plates), and MGIT960 methods, the levels of categorical agreement between the observed and expected results and the level of sensitivity at detecting an isolate as resistant were highest at 0.25, 0.12, 0.12, and 1 μg/ml BDQ concentrations, respectively (Table 2). The levels of categorical agreement were highest for BMD (dry plates) and MGIT960, with both above 99%; the levels of categorical agreement for AP and BMD (frozen plates) were slightly lower at 96.7% and 98.1%, respectively. The very major error rates, defined as wrongly calling an isolate susceptible by the evaluated method when it was resistant by the reference standard, were the lowest at the respective concentrations. The very major error rate was highest for AP at 12.0%, and no major errors were observed for BMD (dry plates) or MGIT960.

**TABLE 2:** Agreement, sensitivity and specificity, and errors for the DST methods^*a*^

Test method (replicates)	CC/BP(μg/ml)	Categorical agreement (%)	Kappa	Sensitivity (%) [95% CI]	Specificity (%) [95% CI]	% of isolates with very major errors (no. of isolates with very major errors/total no. of isolates)	% of isolates with major errors (no. of isolates with major errors/total no. of isolates)
Agar proportion (*n* = 577)	**0.25**	**96.7**	**0.9112**	**88.0 [81.7–92.7]**	**99.8 [98.7–100.0]**	**12.0 (18/150)**	**0.2 (1/427)**
	0.5	88.6	0.6532	56.0 [47.7–64.1]	100.0 [99.1–100.0]	44.0 (66/150)	0
	1	82.0	0.3956	30.7 [23.4–38.7]	100.0 [99.1–100.0]	69.3 (104/150)	0

BMD frozen (*n* = 572)	**0.12**	**98.1**	**0.9484**	**97.8 [93.8–99.6]**	**98.2 [96.4–99.2]**	**2.2 (3/139)**	**1.8 (8/433)**
	0.25	97.6	0.9315	90.6 [84.5–94.9]	99.8 [98.7–100.0]	9.35 (13/139)	0.2 (1/433)

BMD dry (*n* = 584)	**0.12**	**99.3**	**0.9822**	**100.0 [97.6–100.0]**	**99.1 [97.7–99.7]**	**0**	**0.9 (4/434)**
	0.25	97.3	0.9256	89.3 [83.3–93.8]	100.0 [99.2–100.0]	10.7 (16/150)	0

MGIT960 (*n* = 594)	**1**	**99.8**	**0.9956**	**100.0 [97.6–100.0]**	**99.8 [98.8–100.0]**	**0**	**0.2 (1/444)**
	2	93.3	0.8043	73.3 [65.5–80.2]	100.0 [99.2–100.0]	26.7 (40/150)	0

a CC data (indicated in micrograms per milliliter) apply to the agar proportion and MGIT960 methods, and MIC breakpoint data (indicated in micrograms per milliliter) apply to both the frozen and dry microtiter plates using the BMD method. Sensitivity data represent percentages of isolates classified as resistant by the test method against the total number of true resistant isolates. Specificity data represent percentages of isolates classified as susceptible against the total number of true susceptible isolates. Major error, resistant by the evaluated method and susceptible by the reference standard. Very major error, susceptible by the evaluated method and resistant by the reference standard. None of the errors occurred in all three replicates. Rows in bold indicate the concentrations at which the categorical agreement between the observed results and the expected results as well as the sensitivity/specificity at detecting an isolate as susceptible or resistant were highest. BMD, broth microdilution; BP, breakpoint; CC, critical concentration; CI, confidence interval; DST, drug susceptibility testing; MGIT, Mycobacteria Growth Indicator Tube.

### Classification of wild-type population and effects of *Rv0678* and *atpE* mutations at BDQ phenotypic breakpoints.

The CC of 0.25 μg/ml (AP) identified 99.8% of wild-type clinical isolates as BDQ susceptible (only one isolate tested as showing BDQ resistance in one replicate at one laboratory), 85% of *Rv0678* mutants as BDQ resistant, and 100% of *atpE* mutants as BDQ resistant. Use of the BDQ breakpoint of 0.12 μg/ml (BMD) identified 98.2% (BMD frozen plates) and 99.1% (BMD dry plates) of a wild-type population as BDQ susceptible (Table 3). Use of the same breakpoint detected 97.3% (BMD frozen plates) and 100% (BMD dry plates) of *Rv0678* mutants as BDQ resistant, respectively. Both DST methods identified *atpE* mutants as BDQ resistant at the same breakpoint. MGIT960 at the BDQ CC of 1 μg/ml identified nearly 100% of the wild-type isolates as BDQ susceptible and 100% of *Rv0678* and *atpE* mutants as BDQ resistant.

**TABLE 3:** Percentages of wild-type and *Rv0678* and *atpE* mutant isolates classified as susceptible or resistant by the four test methods at BDQ phenotypic breakpoints^*a*^

Test method	BDQ conc	% of M. tuberculosis isolates showing susceptibility or resistance (no. of susceptible or resistant isolates/total no. of isolates)		
		Wild type (susceptible)	*Rv0678* (resistant)	*atpE* (resistant)
Agar proportion	**0.25**	**99.8 (426/427)**	**85.0 (102/120)**	**100.0 (30/30)**
	0.5	100.0 (427/427)	45.0 (54/120)	100.0 (30/30)
	1	100.0 (427/427)	13.3 (16/120)	100.0 (30/30)

BMD frozen	**0.12**	**98.2 (425/433)**	**97.3 (109/112)**	**100.0 (27/27)**
	0.25	99.8 (432/433)	88.4 (99/112)	100.0 (27/27)

BMD dry	**0.12**	**99.1 (430/434)**	**100.0 (120/120)**	**100.0 (30/30)**
	0.25	100.0 (434/434)	86.7 (104/120)	100.0 (30/30)

MGIT960	**1**	**99.8 (443/444)**	**100.0 (120/120)**	**100.0 (30/30)**
	2	100.0 (444/444)	66.7 (80/120)	100.0 (30/30)

a Rows in bold indicate the critical concentration of 0.25 μg/ml using the agar proportion method, BDQ breakpoints of 0.12 μg/ml using the BMD method, and CC of 1 μg/ml using the MGIT960 method. BDQ, bedaquiline; BMD, broth microdilution; CC, critical concentration; MGIT, Mycobacteria Growth Indicator Tube.

### Intralaboratory reproducibility of DST methods.

Comparisons of the three replicates within each laboratory showed that the dry plate and MGIT960 assays were the most reproducible DST methods for BDQ (Table 4). For Lab-1 and Lab-2, the day-to-day reproducibility rates were ≥97% for all isolates, irrespective of the resistance subtype, for all DST methods. For Lab-3, reproducibility rates were lower (87% to 95%) for the BMD frozen and dry plates, mainly due to lower reproducibility in identifying wild-type isolates as BDQ susceptible from day to day (83% to 93%), while the *Rv0678* and *atpE* mutants tested as BDQ resistant as expected (100%). For Lab-4, reproducibility was lower with the AP (87.5%) and BMD frozen plates (71.4%) for the *Rv0678* mutants than for the other isolates. For Lab-5, reproducibility of AP was lower (62.5%) for the *Rv0678* mutants and that of the BMD frozen plates for all subtypes (83.3%), but the reproducibility of BMD dry plate results was 97.3% and of MGIT960 results was 100% overall. Lab-5 also reported a low number of isolates in the replicates due to technical issues (i.e., dried wells in the outer side of some of the BMD frozen plates during incubation).

**TABLE 4:** Intralaboratory reproducibility of DST methods using a CC of 0.25 μg/ml for the agar proportion method, a BDQ breakpoint of 0.12 μg/ml for the BMD method, and a CC of 1 μg/ml for the MGIT960 method

Laboratory	DST method^*a*^	*n*	% of M. tuberculosis isolates with reproducible results			
			All	Wild type	*Rv0678*	*atpE*
Lab-1	Agar proportion	39	100.0	100.0	100.0	100.0
	BMD frozen	39	100.0	100.0	100.0	100.0
	BMD dry	40	100.0	100.0	100.0	100.0
	MGIT960	40	97.5	96.7	100.0	100.0

Lab-2	Agar proportion	40	100.0	100.0	100.0	100.0
	BMD frozen	40	100.0	100.0	100.0	100.0
	BMD dry	40	100.0	100.0	100.0	100.0
	MGIT960	40	100.0	100.0	100.0	100.0

Lab-3	Agar proportion	40	97.5	96.7	100.0	100.0
	BMD frozen	40	87.5	83.3	100.0	100.0
	BMD dry	40	95.0	93.3	100.0	100.0
	MGIT960	40	100.0	100.0	100.0	100.0

Lab-4	Agar proportion	40	97.5	100.0	87.5	100.0
	BMD frozen	38	94.7	100.0	71.4	100.0
	BMD dry	38	100.0	100.0	100.0	100.0
	MGIT960	40	100.0	100.0	100.0	100.0

Lab-5	Agar proportion	32	90.6	100.0	62.5	100.0
	BMD frozen	24	83.3	85.7	66.7	—^*b*^
	BMD dry	37	97.3	96.3	100.0	100.0
	MGIT960	39	100.0	100.0	100.0	100.0

a BMD, broth microdilution; DST, drug susceptibility testing; MGIT, Mycobacteria Growth Indicator Tube.

b —, no valid reproducibility result for Lab-5.

### Interlaboratory reproducibility of DST methods.

The results of the interlaboratory reproducibility assays also showed that BMD using dry plates and MGIT960 were generally the most reproducible DST methods for BDQ (Table 5). For the 7H11 AP method at the CC of 0.25 μg/ml, there was 96.0% agreement across the five laboratories for the results from all isolates in aggregate, with 98.6%, 85.0%, and 100.0% agreement for the wild-type, *Rv0678* mutant, and *atpE* mutant populations, respectively (Table 5). The lower reproducibility rate for *Rv0678* mutants was due to the disagreement between the results from the various laboratories in identifying these mutants as BDQ resistant (Table 3). This was particularly noted for AP, with 85% reproducibility compared with the levels seen with the other methods, which were 97.3% or higher. For the BMD method using the frozen plates, the levels of reproducibility between laboratories were 97.0%, 96.8%, and 97.2% for all isolates, the wild-type isolates, and the *Rv0678* mutant isolates, respectively (Table 5). For BMD using dry plates and MGIT960, high interlaboratory reproducibility rates (close to 100% for the wild-type isolates and 100% for the *Rv0678* and *atpE* mutant isolates) were seen for all isolates in the aggregate and for all resistance subtypes (Table 5), which is consistent with the high sensitivity and low error rates (Table 2) and with each laboratory categorizing wild-type isolates as BDQ susceptible and *Rv0678* and *atpE* mutants as BDQ resistant (Table 3).

**TABLE 5:** Interlaboratory reproducibility of DST methods^*a*^

DST methods	CC/BP	N	All (%)	Wild type (%)	*Rv0678* (%)	*atpE* (%)
Agar proportion	0.25	577	96.0	98.6	85.0	100.0
BMD frozen	0.12	572	97.0	96.8	97.2	100.0
BMD dry	0.12	584	99.5	99.3	100.0	100.0
MGIT960	1	594	99.8	99.8	100.0	100.0

a Critical concentration data (indicated in micrograms per milliliter) apply to the agar proportion and MGIT960 methods, and MIC breakpoint data (indicated in micrograms per milliliter) apply to both frozen and dry microtiter plates using the BMD method. BMD, broth microdilution; BP, breakpoint; CC, critical concentration; DST, drug susceptibility testing; MGIT, Mycobacteria Growth Indicator Tube.

### Final validation of the BDQ DST methods and interpretive criteria.

The final validated BDQ DST methods and interpretive criteria are summarized in Table 6. The strains classified as resistant were those with a MGIT growth unit (GU) value of >100 in the drug-containing tube at the concentration of 1 mg/ml, a BMD MIC of ≥0.25 μg/ml, or an AP of ≥1% at the concentration of 0.25 mg/ml.

**TABLE 6:** BDQ DST methods and interpretive criteria^*a*^

DST method	CC/BP (μg/ml)	Value for indicated interpretive criterion	
		Susceptible	Resistant
MGIT	1^*b*^	GU ≤ 100	GU > 100
7H9 broth MIC (BMD)	0.12^*c*^	≤0.12 μg/ml	≥0.25 μg/ml
Agar proportion	0.25^*d*^	<1%	≥1%

a BDQ, bedaquiline; BP, breakpoint; CC, critical concentration; DST, drug susceptibility testing; GU, growth unit.

b Critical concentration.

c MIC breakpoint; applies to both frozen and dry microtiter plates.

d Critical concentration; applies to both 7H10 agar and 7H11 agar.

## DISCUSSION

With increasing use of BDQ, reports of clinical relapses associated with drug resistance and cross-resistance with clofazimine have emerged (30–33), quelling the early excitement based on improving treatment outcomes. Hence, more-stringent measures are required to control the emergence of BDQ resistance, including systematic surveillance of drug resistance and rapid and reliable DST to personalize anti-TB treatment.

Availability of reliable interpretive criteria for BDQ DST results is important to many stakeholders, including the company/nonprofit organization holding the marketing approval, the WHO, the FDA, the CLSI, European Medicines Agency (EMA), and the European Committee on Antimicrobial Susceptibility Testing (EUCAST). Prior to confirming DST with interpretive criteria, the EUCAST and FDA set provisional epidemiological cutoff values (ECVs) and clinical breakpoints for BDQ (34), while the WHO issued interim CCs for BDQ (23) and published requirements for drug susceptibility testing of anti-TB medicines (23). The intent in our study was therefore to reconcile requirements from the key stakeholders by addressing the gap in the knowledge related to the three methods and associated interpretive criteria for resistance determination by applying standardized procedures.

This multicountry, multilaboratory EQA study validated the sensitivity and specificity of three methods using provisional BDQ MIC breakpoints (1 μg/ml for the MGIT method and 0.12 μg/ml for the BMD method reported by Ismail et al. [15] and the WHO interim CCs of 1 μg/ml for MGIT and 0.25 μg/ml for the 7H11 AP method [17, 23]). For the AP method, BMD (frozen or dry plates), and MGIT960, the categorical agreement between the observed and expected results, and their sensitivity/specificity at detecting an isolate as resistant or susceptible, were highest at the 0.25, 0.12, and 1 μg/ml BDQ concentrations, respectively, while error rates for wrongly calling an isolate susceptible were lowest at these concentrations. The most highly reproducible DST methods for BDQ were BMD using dry plates and MGIT960.

Epidemiological cutoff values (ECVs) are commonly used for clinical breakpoint setting, providing a basis to define susceptibility. The BDQ concentrations tested in this EQA study were selected based on previous findings (15; internal communication, DREAM Interim Report, 2018). Since concentrations lower than the ECVs split the wild-type MIC distributions, there was little value in testing concentrations below the ECVs in this EQA study; hence, higher concentrations were tested to ensure that the correct breakpoints were not missed. Indeed, the breakpoints used were able to identify the wild-type population as BDQ susceptible and the *Rv0678* and *atpE* mutants as BDQ resistant, correctly identifying 97% of the isolates by the BMD frozen, BMD dry, and MGIT 960 methods. However, use of the AP assay at the WHO-recommended CC of 0.25 μg/ml resulted in detection of the wild-type population as susceptible to BDQ, while only the *Rv0678* mutants with high MICs and *atpE* mutants were detected as resistant to BDQ. The AP assay used with a CC of 0.25 μg/ml would therefore not adequately detect resistant M. tuberculosis isolates harboring *Rv0678* mutations at close to the CC.

From our findings, employment of the three BDQ phenotypic DST methods can be recommended as follows: BMD using dry plates and MGIT960 should be recommended as the preferred phenotypic DST methods for BDQ, while the AP assay should be used only to rule in susceptible isolates when MGIT960 or BMD using dry microtiter plates is unavailable.

The EQA study included five laboratories highly experienced in M. tuberculosis DST and located in geographically diverse countries. The testing of 20 strains in each laboratory, in addition to the use of conditions whereby each study investigator was blind to the identities of the others, means that the findings are likely to be globally representative. Validating the methods and interpretive criteria in a standardized manner across countries further provides robust confirmation of the precedent work. However, the study was limited in that there were no U.S. data and one laboratory (Lab-5) reported a low number of isolates in the replicates for BMD frozen plates, due to technical issues (dried-out wells on the border of some of the MIC plates during incubation).

The findings from this EQA study should provide standardization of DST methodology and interpretative criteria to facilitate routine phenotypic BDQ DST. The totality of the data generated from this study will inform breakpoint-setting bodies (i.e., the U.S. FDA and CLSI, WHO, EMA, and EUCAST) to set or revise interpretive criteria for BDQ phenotypic DST and may also support regulatory clearance of *in vitro* DST devices such as MGIT960 and dry microtiter plates (24). EUCAST recently released the protocol to be used for validation of all DST methods for M. tuberculosis; the next step will be the comparison of our data with the standard methodology. BDQ is now considered a first-line treatment for RR/MDR-TB (35), and it is likely that BDQ-based regimens will be required for treatment of an increasing number of drug-resistant patients. The availability of reliable BDQ DST methodologies is thus critical for detection and monitoring of the emergence of BDQ resistance. Such DST primarily uses methodologies already in use for TB DST, facilitating its implementation, and the availability of BDQ addresses an important gap in the management of TB.
